# Effectiveness & safety of semaglutide in weight reduction in obese nondiabetic patients: A systematic review and meta-analysis

**DOI:** 10.1097/MD.0000000000049986

**Published:** 2026-07-31

**Authors:** Faryal Naz, Fawad Qaiser, Arif Mumtaz, Siraj-ud Din, Amjad Mustafa, Asif Ullah, Abida Perveen, Jahanzeb Malik

**Affiliations:** a Department of Medicine, Ibn e Seena Hospital, Kabul, Afghanistan.

**Keywords:** nondiabetic patients, obesity, randomized controlled trials, semaglutide, weight loss

## Abstract

**Background:**

Obesity is a chronic multifactorial disease characterized by excess adiposity and persistent low-grade systemic inflammation, contributing substantially to cardiometabolic morbidity and mortality. Semaglutide, a long-acting glucagon-like peptide-1 receptor agonist approved for chronic weight management, promotes weight loss by reducing appetite, delaying gastric emptying, and decreasing energy intake while also demonstrating potential anti-inflammatory effects. Although semaglutide has shown promising efficacy for weight reduction, an updated evaluation of its efficacy and safety in nondiabetic individuals with overweight or obesity is warranted.

**Methods:**

A systematic review and meta-analysis was conducted according to PRISMA 2020 guidelines. PubMed/MEDLINE, Embase, Scopus, and CINAHL were searched from inception to March 2025 for randomized controlled trials evaluating subcutaneous semaglutide in adults with overweight or obesity without type 2 diabetes mellitus. The primary outcome was the percentage change in body weight from baseline. Secondary outcomes included overall gastrointestinal adverse events, nausea, vomiting, diarrhea, constipation, treatment discontinuation due to adverse events, and serious adverse events. Pooled estimates were calculated using a random-effects model.

**Results:**

Four randomized controlled trials involving 3613 participants met the eligibility criteria. Semaglutide produced significantly greater weight loss than placebo (mean difference: −11.85%; 95% confidence interval [CI]: −12.81 to − 10.90; *P* < .00001). Overall gastrointestinal adverse events were significantly more frequent with semaglutide, with nausea, vomiting, diarrhea, and constipation representing the most commonly reported events. Treatment discontinuation due to adverse events was also significantly higher in the semaglutide group (RR 2.62, 95% CI: 1.70–4.03; *P* = .001). Serious adverse events, including acute pancreatitis and cholelithiasis, were uncommon.

**Conclusions:**

Subcutaneous semaglutide is an effective treatment for weight reduction in adults with overweight or obesity without type 2 diabetes mellitus but is associated with an increased risk of gastrointestinal adverse events, particularly nausea, vomiting, diarrhea, and constipation, as well as higher treatment discontinuation. Serious adverse events were rare. Further long-term randomized trials are needed to evaluate the durability of weight loss and the long-term safety profile of semaglutide.

## 1. Introduction

Obesity is a chronic, relapsing, multifactorial disease characterized by excessive adipose tissue accumulation that substantially increases the risk of type 2 diabetes mellitus (T2DM), cardiovascular disease, musculoskeletal disorders, several malignancies, and premature mortality.^[1]^ It results from a complex interaction of genetic, metabolic, behavioral, and environmental factors that disrupt the balance between energy intake and expenditure. The global burden of obesity has increased dramatically over recent decades, with its prevalence nearly tripling since 1975. By 2016, more than 1.9 billion adults were overweight, including over 650 million classified as obese, making obesity one of the leading contributors to global morbidity and healthcare expenditure.^[2]^ Consequently, effective and sustainable therapeutic strategies remain a major public health priority.

Current obesity management includes lifestyle modification, behavioral interventions, pharmacotherapy, and bariatric surgery.^[3,4]^ Although dietary interventions and increased physical activity remain the cornerstone of treatment, long-term weight maintenance is often difficult because of adaptive physiological mechanisms that promote weight regain. Pharmacological therapies, therefore, play an increasingly important role as adjuncts to lifestyle modification. However, currently approved anti-obesity medications are limited, and several agents, including phentermine, phentermine/topiramate, and bupropion/naltrexone, are associated with adverse cardiovascular, neurological, or psychiatric effects that may limit their long-term use.^[5]^

Semaglutide is a long-acting glucagon-like peptide-1 receptor agonist (GLP-1 RA) originally developed for the treatment of T2DM and subsequently approved for chronic weight management. By activating GLP-1 receptors, semaglutide enhances glucose-dependent insulin secretion, suppresses glucagon release, delays gastric emptying, and acts on hypothalamic appetite-regulating centers to increase satiety and reduce caloric intake.^[6-8]^ Compared with earlier GLP-1 receptor agonists, semaglutide possesses favorable pharmacokinetic properties, including a prolonged half-life permitting once-weekly administration, which improves treatment adherence. Clinical trials have consistently demonstrated greater weight reduction with semaglutide than with several other GLP-1 receptor agonists, including liraglutide, supporting its emergence as one of the most effective pharmacological treatments for obesity.^[9-11]^

Beyond weight reduction, obesity is increasingly recognized as a chronic state of low-grade systemic inflammation characterized by adipose tissue dysfunction, immune cell activation, and excessive production of pro-inflammatory cytokines. This inflammatory environment contributes to insulin resistance, endothelial dysfunction, cardiovascular disease, and other obesity-related complications. Experimental evidence further suggests that obesity-associated metabolic inflammation is linked to impaired autophagy and autolysosomal dysfunction, thereby perpetuating metabolic disturbances.^[12]^ Emerging preclinical studies indicate that semaglutide may exert anti-inflammatory effects through activation of the AMPK/SIRT1 signaling pathway and inhibition of NF-κB-mediated inflammatory responses, suggesting that its therapeutic benefits may extend beyond appetite suppression and weight loss.^[13]^

Several randomized controlled trials have demonstrated the efficacy of semaglutide in promoting clinically meaningful weight loss in adults with overweight or obesity without diabetes. Furthermore, previous systematic reviews and meta-analyses by Tan et al^[14]^ and Gao et al^[15]^ concluded that semaglutide significantly reduces body weight while maintaining an acceptable safety profile. However, these reviews were conducted using evidence available through 2022 and included a relatively limited number of randomized trials. Since then, additional high-quality randomized controlled trials with longer follow-up durations and more comprehensive reporting of efficacy and safety outcomes have become available. Consequently, an updated synthesis of the evidence is warranted to provide more precise estimates of treatment effects and to better characterize clinically relevant adverse events, including gastrointestinal complications, treatment discontinuation, and serious adverse events.

Therefore, the present systematic review and meta-analysis aimed to comprehensively evaluate the efficacy and safety of subcutaneous semaglutide in adults with overweight or obesity without T2DM by incorporating all eligible randomized controlled trials published through March 2025. By providing an updated quantitative synthesis of both efficacy and safety outcomes, this review seeks to strengthen the current evidence base and inform clinical decision-making regarding the use of semaglutide for obesity management.

### 1.1. Objectives

This study aimed to conduct a systematic review and meta-analysis of randomized controlled trials (RCTs) that investigated the use of subcutaneous semaglutide in obese patients without type 2 diabetes mellitus (T2DM). The primary objective was to evaluate the percentage of weight loss from baseline after semaglutide treatment. Additionally, the study aimed to analyze the risk of gastrointestinal side effects, the rate of treatment discontinuation, and the incidence of serious adverse events associated with semaglutide.

## 2. Materials and methods

### 2.1. Protocol and registration

This systematic review and meta-analysis was conducted in accordance with the PRISMA 2020 guidelines. The review protocol was not prospectively registered in PROSPERO or any other systematic review registry.

### 2.2. Search strategy

This systematic review and meta-analysis was conducted in accordance with the Preferred Reporting Items for Systematic Reviews and Meta-Analyses (PRISMA 2020) guidelines. A comprehensive literature search was independently performed by 2 reviewers (HCT and MMM) in PubMed/MEDLINE, Embase, Scopus, and CINAHL from database inception to March 2025 to identify randomized controlled trials evaluating the efficacy and safety of subcutaneous semaglutide in adults with obesity without type 2 diabetes mellitus (T2DM). The search strategy combined controlled vocabulary (Medical Subject Headings [MeSH] and Emtree terms where applicable) with free-text keywords and Boolean operators. The following search terms and their synonyms were used in various combinations: “semaglutide,” “GLP-1 receptor agonist,” “glucagon-like peptide-1 receptor agonist,” “obesity,” “overweight,” “adiposity,” “weight loss,” “weight reduction,” “weight management,” and “anti-obesity therapy.” A representative PubMed search string was: (“Semaglutide”[Mesh] OR semaglutide OR “GLP-1 receptor agonist*” OR “glucagon-like peptide-1 receptor agonist*”) AND (“Obesity”[Mesh] OR obesity OR overweight OR adiposity OR “weight loss” OR “weight reduction” OR “weight management” OR “anti-obesity therapy”). No language or publication-year restrictions were applied during the initial search. In addition, the reference lists of eligible studies and relevant review articles were manually screened to identify potentially relevant studies missed by the electronic search. Following duplicate removal, titles and abstracts were independently screened by 2 reviewers, and potentially eligible articles underwent full-text assessment. Any disagreements regarding study eligibility were resolved through discussion and consultation with a third reviewer (OAD) until consensus was achieved.

### 2.3. Eligibility criteria

Studies were selected according to the PICOS (Population, Intervention, Comparison, Outcomes, and Study Design) framework. Eligible studies were randomized controlled trials (RCTs) enrolling adults (≥18 years) with overweight or obesity and without a diagnosis of type 2 diabetes mellitus (T2DM). Studies evaluating subcutaneous semaglutide at any approved dose for weight management were included. The comparator group consisted of placebo. Eligible studies were required to report at least 1 predefined efficacy or safety outcome, including percentage change in body weight, gastrointestinal adverse events, treatment discontinuation due to adverse events, or serious adverse events. Only full-text articles published in peer-reviewed journals were considered for inclusion. Studies were excluded if they involved participants with T2DM, lacked a placebo comparator, did not report relevant outcome data, were review articles, meta-analyses, editorials, conference abstracts, case reports, preprints, observational studies, or duplicate publications. Ongoing trials and studies without complete published outcome data were screened but excluded from both the qualitative and quantitative analyses.

### 2.4. Interventions and outcomes

The intervention of interest was subcutaneous semaglutide administered for weight management in adults with overweight or obesity without type 2 diabetes mellitus. The primary outcome was the percentage change in body weight from baseline following semaglutide treatment compared with placebo. Secondary outcomes included gastrointestinal adverse events, treatment discontinuation due to adverse events, and serious adverse events. Only placebo-controlled randomized controlled trials reporting at least 1 of the predefined efficacy or safety outcomes were eligible for inclusion in the review and meta-analysis.

### 2.5. Selection of studies

Two reviewers independently screened titles, abstracts, and full-text articles according to the predefined eligibility criteria. A total of 54 records were identified through database searching. After the removal of 35 duplicate records, 19 studies underwent title and abstract screening. Four studies were excluded at this stage because they were abstracts only, contained incomplete data, or did not report the primary outcome of interest. Full texts of 11 potentially eligible studies were retrieved and assessed for eligibility. Five studies were subsequently excluded because they did not include a comparison for the primary outcome, while 2 preprints were excluded because they had not undergone peer review. Ultimately, 4 randomized controlled trials met the inclusion criteria and were included in the systematic review and meta-analysis. Any disagreements regarding study selection were resolved through discussion and consultation with a third reviewer until consensus was achieved.

### 2.6. Data extraction and risk of bias assessment

The selected articles were downloaded and independently reviewed by the reviewers. Any discrepancies were resolved through discussion and consensus, with input from a third expert investigator when needed. A data collection form was used to extract information from each article, including the author, study population demographics, inclusion and exclusion criteria, intervention and comparison methods, the primary outcome of percent weight reduction, and gastrointestinal adverse events. The quality of the studies was assessed using the Cochrane Collaboration’s tool for evaluating the risk of bias. This assessment considered factors such as random sequence generation, allocation concealment, blinding, incomplete outcome data, selective reporting, and other biases, rating them as high, low, or unclear. Discrepancies were resolved through discussion.

### 2.7. Data synthesis and analysis

Data synthesis and statistical analyses were performed using Review Manager (RevMan) version 5.4 for Mac. Continuous outcomes were pooled as mean differences (MDs) with corresponding 95% confidence intervals (CIs), while dichotomous outcomes were summarized using risk ratios (RRs) and 95% CIs. A 2-sided *P*-value < .05 was considered statistically significant. Given the anticipated clinical and methodological heterogeneity across studies, including variations in semaglutide dose, treatment duration, participant characteristics, obesity severity, and lifestyle intervention protocols, a random-effects model was applied a priori for all analyses. Statistical heterogeneity was assessed using Cochran *Q* test and quantified using the *I*^2^ statistic, with values of 25%, 50%, and 75% representing low, moderate, and high heterogeneity, respectively. Sensitivity analyses were planned to evaluate the robustness of the pooled estimates by assessing the influence of individual studies and reviewing study eligibility and methodological characteristics. However, owing to the limited number of included studies, formal subgroup analyses, meta-regression, and publication bias assessments were not performed. Ethical approval was not required because this study was a systematic review and meta-analysis of previously published data.

## 3. Results

### 3.1. Study selection

**Figure 1.**
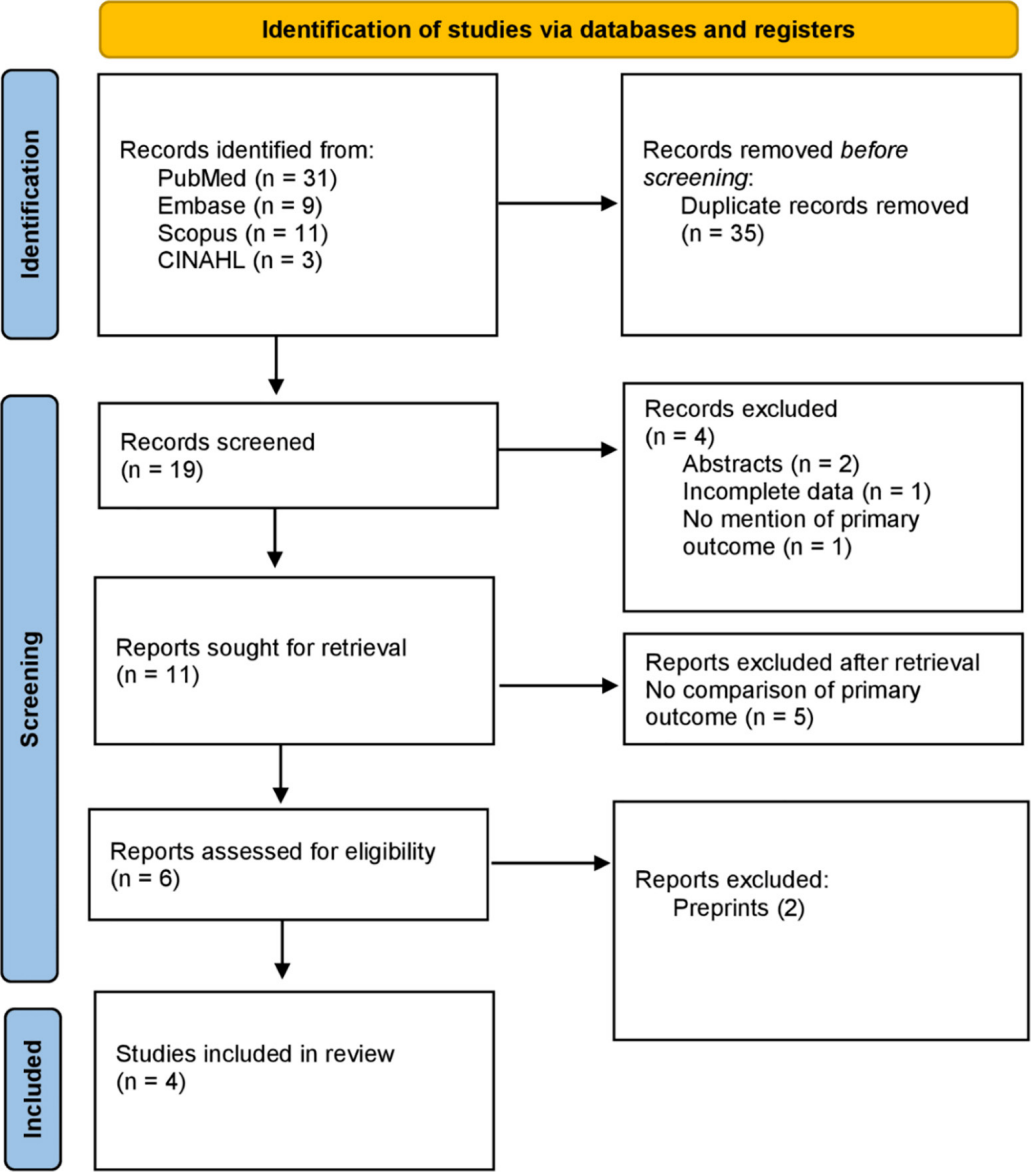
PRISMA flowchart. PRISMA = Preferred Reporting Items for Systematic Reviews and Meta-Analyses.

The search identified a total of 54 articles. After eliminating 35 duplicates, 8 articles were excluded based on their titles and abstracts. This left 11 full-text articles to be evaluated for eligibility. Of these, 5 were excluded for reasons such as involving patients with diabetes, nonobese populations, using interventions other than semaglutide, or having outcomes that did not align with the review’s objectives. Ultimately, 4 randomized controlled trials (RCTs) were included in the analysis. These trials assessed the percentage change in body weight after treatment with semaglutide compared to placebo and reported common adverse effects related to the treatment. No similar ongoing studies were found in the search, as shown in Figure 1.

### 3.2. Study characteristics

The analysis included 3613 participants across the 4 trials, with 2350 individuals in the semaglutide group and 1263 in the placebo group. Baseline characteristics were comparable across both groups in all 4 trials described in Table 1. Participants were adults aged 18 or older with a BMI of 30 kg/m^2^ or higher, or a BMI of 27 kg/m^2^ or higher with at least 1 weight-related comorbidity, excluding those with diabetes. Common comorbidities among participants included hypertension and dyslipidemia.

**Table 1:** Patient baseline characteristics.

First author, year	Mean weight (kg) – Semaglutide group	Mean weight (kg) – Placebo group	Mean BMI (kg/m^2^) – Semaglutide group	Mean BMI (kg/m^2^) – Placebo group	Mean age (yr) – Semaglutide group	Mean age (yr) – Placebo group	Female sex (%) – Semaglutide group	Female sex (%) – Placebo group
O’Neil, 2018	113.2	114.2	39.9	40.1	48	46	66/102 (65%)	88/136 (65%)
Rubino, 2021	96.5	95.4	34.5	34.1	47	46	429/535 (80.2%)	205/268 (76.5%)
Wadden, 2021	106.9	103.7	38.1	37.8	46	46	315/407 (77.4%)	180/204 (88.2%)
Wilding, 2021	105.4	105.2	37.8	38	46	47	955/1306 (73.1%)	498/655 (76%)

BMI = body mass index.

In the study by Wilding et al., participants received a weekly subcutaneous injection of semaglutide, starting at 0.25 mg and increasing every 4 weeks until reaching a target dose of 2.4 mg. In contrast, Rubino’s study randomized participants to either continue with semaglutide or switch to a placebo after reaching the target dose. O’Neil et al administered a smaller daily dose of semaglutide, ranging from 0.05 mg to 0.4 mg. The treatment duration varied slightly among the studies: Wilding, Rubino, and Wadden conducted their studies over 68 weeks, while O’Neil’s study lasted 52 weeks. All trials measured the percent change in body weight from baseline to the end of the study and reported the most common adverse events associated with the treatment shown in Table 2.

**Table 2:** Study characteristics.

First author, year	Study design	Study population	Inclusion criteria	Exclusion criteria	Interventions	Outcome
O’Neil, 2018	RCT	≥18 yr old with BMI ≥ 30 kg/m^2^	BMI ≥ 30 kg/m^2^ with no weight fluctuation > 5 kg in the 90 d before screening; At least 1 unsuccessful nonsurgical weight-loss attempt; free from major depressive symptoms	Diabetes	Semaglutide injected subcutaneously once daily at doses (0.05 mg, 0.1 mg, 0.2 mg, 0.3 mg, 0.4 mg) or liraglutide (3.0 mg), with doses starting at 0.05 mg and incrementally escalated every 4 weeks to the next level until reaching final doses vs placebo of equal injection volume	Percent change in body weight from baseline to week 52; most common reported adverse events
Rubino, 2021	RCT	≥18 yr old with BMI ≥ 30 kg/m^2^ or BMI ≥ 27 kg/m^2^ with at least 1 treated or untreated weight-related comorbidity	At least 1 self-reported unsuccessful dietary effort to lose weight; BMI of 27 kg/m^2^ or higher with at least 1 treated or untreated weight-related comorbidity (hypertension, dyslipidemia, obstructive sleep apnea, cardiovascular disease)	Diabetes; HbA1c 6.5% or greater; more than 5 kg change in body weight within 90 d of screening	Semaglutide started at 0.25 mg given subcutaneously once weekly, increased every 4 wk until 2.4 mg by week 16, and continued to week 20, then randomized to continue semaglutide or switch to matching placebo for 48 wk plus lifestyle intervention with monthly counseling, reduced calorie diet, increased physical activity	Percent change in body weight from randomization (week 20) to week 68; most common reported adverse events
Wadden, 2021	RCT	≥18 yr old with BMI ≥ 30 kg/m^2^ or BMI ≥ 27 kg/m^2^ with at least 1 treated or untreated weight-related comorbidity	One or more unsuccessful dietary efforts to lose weight; BMI of 27 kg/m^2^ with at least 1 weight-related comorbidity (hypertension, dyslipidemia, obstructive sleep apnea, cardiovascular disease)	Diabetes; HbA1c 6.5% or greater; More than 5 kg change in body weight within 90 d of screening prior or planned obesity treatment with surgery or a weight loss device	Semaglutide started at 0.25 mg given subcutaneously once weekly, with dose escalation every 4 wk until reaching target dose of 2.4 mg by week 16, continued until week 68 plus diet modification vs placebo	Percent change in body weight by week 68; most common reported adverse events
Wilding, 2021	RCT	≥18 yr old with BMI ≥ 30 kg/m^2^ or BMI ≥ 27 kg/m^2^ with at least 1 treated or untreated weight-related comorbidity	One or more unsuccessful dietary efforts to lose weight; BMI of 27 kg/m^2^ with at least 1 weight-related comorbidity (hypertension, dyslipidemia, obstructive sleep apnea, cardiovascular disease)	Diabetes; HbA1c 6.5% or greater; History of chronic pancreatitis, acute pancreatitis within 180 d before enrollment, previous surgical obesity treatment, and use of anti-obesity medication within 90 d before enrollment	Semaglutide started at 0.25 mg given subcutaneously once weekly, with dose escalation every 4 wk until reaching target dose of 2.4 mg by week 16, continued until week 68 plus counseling sessions every 4 wk on adhering to a reduced calorie diet and increased physical activity vs placebo	Percent change in body weight from baseline to week 68; most common reported adverse events

BMI = body mass index, RCT = randomized controlled trial.

### 3.3. Risk of bias

**Figure 2.**
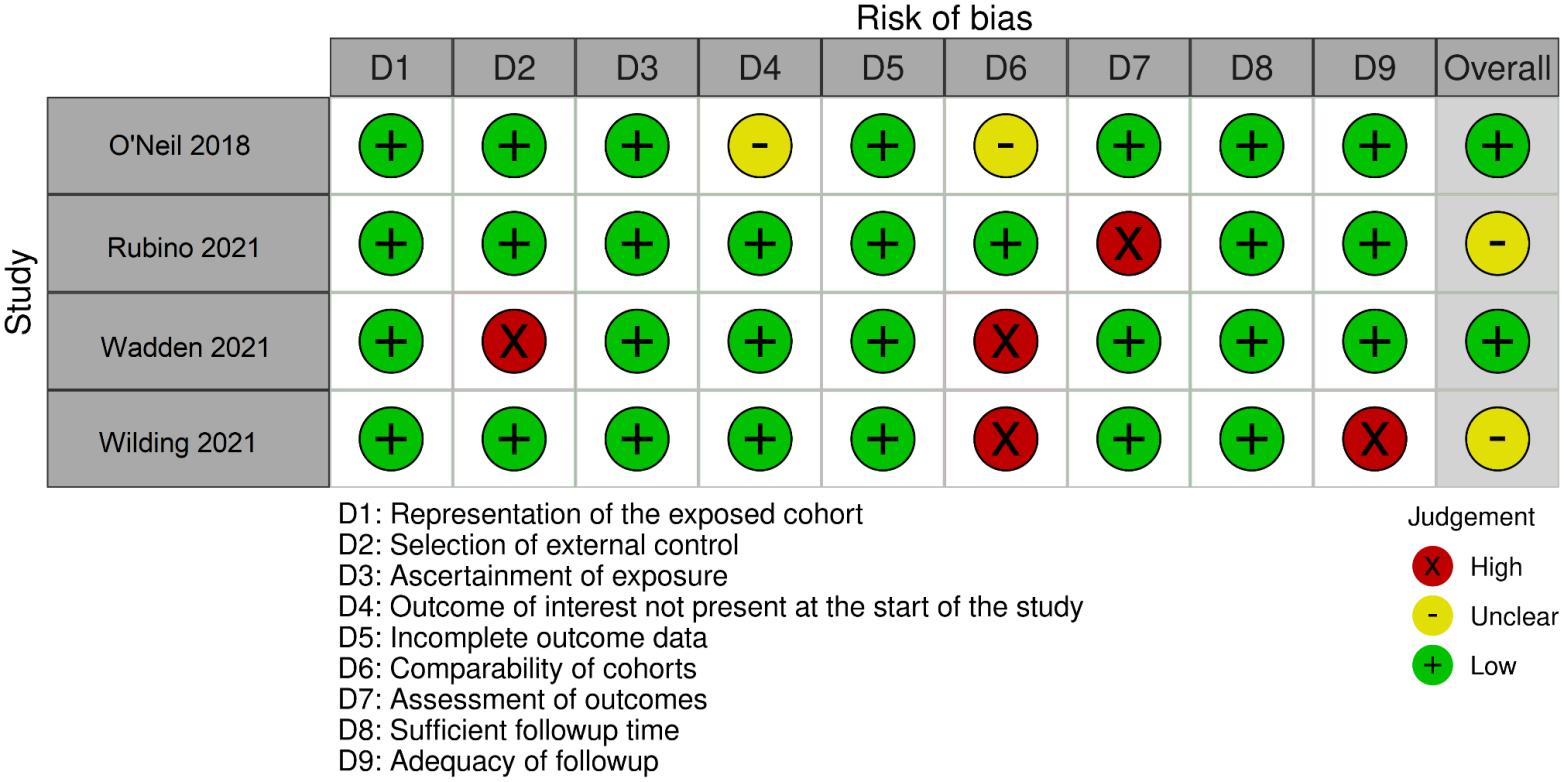
Quality assessment.

The risk of bias summary is shown in Figure 2. Overall, the studies had a generally low risk of bias. However, the risk of attrition bias was considered questionable because all 4 studies included data from participants who were lost to follow-up, which could affect the calculation of the mean weight difference. Other potential biases were also considered questionable due to factors such as adherence to diet and exercise, which could significantly influence weight loss results. All trials were double-blinded and randomized using an interactive web-based response system, and identical-looking placebos and semaglutide injections were utilized to minimize selection, detection, and performance biases.

### 3.4. Outcomes of meta-analysis

#### 3.4.1. Weight reduction

Across the 4 trials, which included 3613 participants (2350 in the semaglutide group and 1263 in the placebo group), semaglutide showed a significant benefit in weight reduction. The mean difference in weight loss between the semaglutide and placebo groups was −11.85%, favoring semaglutide (95% CI [−12.81, −10.90], *P* < .00001). However, the trials had moderate heterogeneity (*I*^2^ = 43%). A sensitivity analysis, excluding the Wadden et al study, reduced heterogeneity to 0%. The Wadden et al study had a unique protocol, prescribing a very low-calorie diet (1000–1200 kcal/d initially, increasing to 1200–1800 kcal/d) and a higher physical activity regimen (starting at 100 min/wk and increasing to 200 min/wk). This protocol may have resulted in greater weight loss in the placebo group, thus reducing the observed mean weight difference as described in Table 3.

**Table 3:** Effect of semaglutide on mean weight difference versus placebo.

Study or subgroup	Semaglutide mean (SD)	Semaglutide total	Placebo mean (SD)	Placebo total	Weight	Mean difference (IV, random, 95% CI)
O’Neil 2018	−13.8 (8.38)	102	−2.3 (8.63)	136	14.50%	−11.50 (−13.68, −9.32)
Rubino 2021	−17.4 (9.2)	535	−5 (9.2)	268	27.10%	−12.40 (−14.75, −10.05)
Wadden 2021	−16 (10.11)	407	−5.7 (10.11)	204	20.50%	−10.30 (−12.00, −8.60)
Wilding 2021	−14.85 (9.91)	1306	−2.41 (9.91)	655	37.90%	−12.44 (−13.37, −11.51)
Total (95% CI)		2350		1263	100.00%	−11.85 (−12.81, −10.90)

CI = confidence interval, SD = standard deviation.

#### 3.4.2. Gastrointestinal adverse events

The review indicated a significantly higher risk of gastrointestinal adverse events (nausea, vomiting, diarrhea, constipation) with semaglutide treatment compared to placebo, as summarized in Table 4. The risk was 1.62 times higher with semaglutide (RR 1.62, 95% CI [1.45, 1.82], *P* < .00001), although there was substantial heterogeneity (*I*^2^ = 81%). Sensitivity analysis reduced heterogeneity to 68%. The primary source of heterogeneity was the dose variation: Rubino, Wadden, and Wilding et al studies administered 2.4 mg once weekly, whereas O’Neil et al used 0.4 mg once weekly. Despite the statistical significance of gastrointestinal adverse events, these events were generally short-lived, transient, and did not necessitate treatment discontinuation.

**Table 4:** Risk of gastrointestinal adverse events with semaglutide treatment versus placebo.

Study or subgroup	Experimental events	Experimental total	Control events	Control total	Weight	Risk ratio (M-H, random, 95% CI)
O’Neil 2018	84	102	52	136	20.20%	2.15 (1.71, 2.72)
Rubino 2021	224	535	70	268	20.70%	1.60 (1.28, 2.01)
Wadden 2021	337	407	129	204	28.70%	1.31 (1.17, 1.47)
Wilding 2021	969	1306	314	655	30.40%	1.55 (1.42, 1.69)
Total (95% CI)	1614	2350	565	1263	100.00%	1.62 (1.45, 1.82)

CI = confidence interval.

#### 3.4.3. Discontinuation due to adverse events

**Figure 3.**
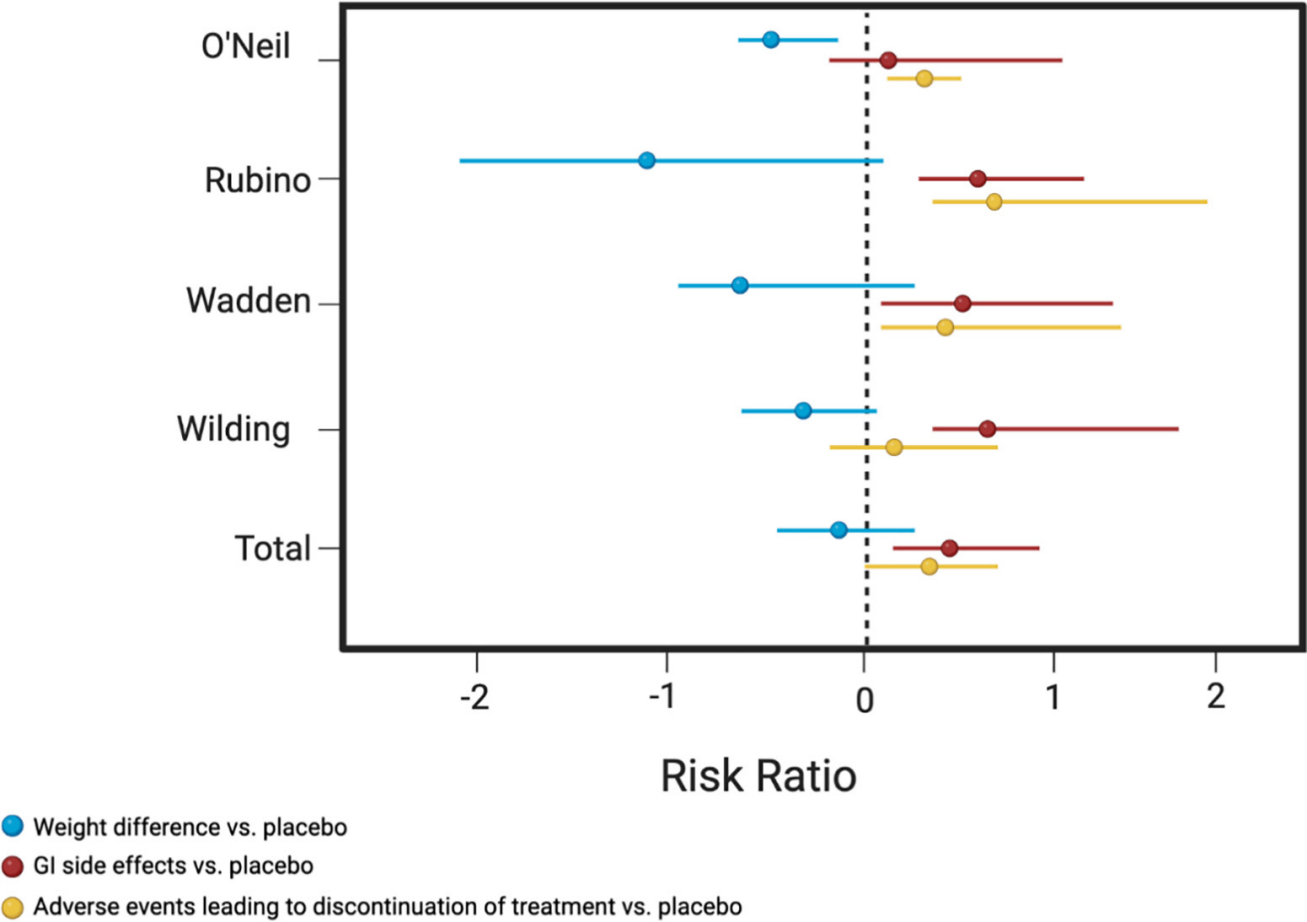
Forest plot showing the effect of semaglutide on mean weight difference versus placebo; the risk of gastrointestinal adverse events with semaglutide treatment versus placebo; and the risk of adverse events leading to discontinuation of treatment with semaglutide versus placebo.

Participants in the semaglutide group were more likely to discontinue treatment due to adverse events as shown in Table 5 with a risk ratio of 2.62 (95% CI [1.70, 4.03], *P* = .001, *I*^2^ = 32%). The discontinuation rate was 6% in the semaglutide group compared to 2.9% in the placebo group, as shown in Figure 3.

**Table 5:** Risk of adverse events leading to discontinuation of treatment with semaglutide versus placebo.

Study or subgroup	Semaglutide events	Semaglutide total	Placebo events	Placebo total	Weight	Risk ratio (M-H, random, 95% CI)
O’Neil 2018	15	102	4	136	15.90%	5.00 (1.71, 14.62)
Rubino 2021	13	535	6	268	19.00%	1.09 (0.42, 2.82)
Wadden 2021	24	407	6	204	21.50%	2.00 (0.83, 4.83)
Wilding 2021	92	1306	20	655	43.60%	2.31 (1.44, 3.71)
Total (95% CI)	144	2350	36	1263	100.00%	2.62 (1.70, 4.03)

CI = confidence interval.

## 4. Discussion

The present systematic review and meta-analysis of 4 randomized controlled trials involving 3613 nondiabetic individuals with obesity demonstrated that subcutaneous semaglutide is highly effective in promoting weight loss, resulting in an average reduction of 11.85% compared with placebo. In addition to its substantial weight-loss efficacy, semaglutide was associated with an increased risk of gastrointestinal adverse events and treatment discontinuation due to adverse events, although serious adverse events remained uncommon. These findings support the role of semaglutide as an effective pharmacological option for obesity management in individuals without T2DM.

The magnitude of weight loss observed in this analysis exceeds the minimum 5% to 10% reduction recommended by current clinical guidelines to achieve meaningful metabolic and cardiovascular benefits.^[14]^ Previous studies have demonstrated that modest weight loss is associated with improvements in insulin sensitivity, glycemic control, blood pressure, lipid profiles, and overall cardiometabolic risk. Therefore, the approximately 12% reduction in body weight observed with semaglutide is likely to translate into clinically significant health benefits beyond weight reduction alone.^[15,16]^ Furthermore, obesity is increasingly recognized as a chronic inflammatory condition characterized by persistent low-grade systemic inflammation. Emerging evidence suggests that GLP-1 receptor agonists may exert anti-inflammatory effects in addition to their effects on appetite regulation and weight reduction, potentially contributing to broader metabolic improvements.

The substantial efficacy observed across the included trials is biologically plausible given the mechanism of action of semaglutide. By activating GLP-1 receptors, semaglutide delays gastric emptying, enhances satiety, reduces appetite, and lowers overall caloric intake. These mechanisms contribute to sustained weight reduction and may explain the consistency of treatment effects observed across the included studies. All participants had previously attempted weight loss with limited success, highlighting the potential value of semaglutide in individuals who fail to achieve adequate weight reduction through lifestyle modification alone.

Our findings are consistent with previous clinical trials demonstrating significant weight reduction with semaglutide therapy.^[17-20]^ Notably, the trial by Rubino et al differed from the other included studies because participants initially received semaglutide before being randomized either to continue treatment or switch to placebo. This design provided important evidence regarding treatment maintenance and suggested that discontinuation of semaglutide may result in partial weight regain, emphasizing the chronic nature of obesity and the potential need for long-term treatment strategies.

The substantial weight loss observed with semaglutide is attributable to multiple complementary mechanisms of action. As a long-acting glucagon-like peptide-1 receptor agonist (GLP-1 RA), semaglutide activates GLP-1 receptors within the hypothalamus and brainstem, particularly in regions involved in appetite regulation, resulting in enhanced satiety, reduced hunger, diminished food cravings, and lower overall energy intake. In addition, semaglutide delays gastric emptying, prolonging gastric distension and promoting early satiety, while improving glucose homeostasis through glucose-dependent insulin secretion and suppression of glucagon release. Beyond these metabolic effects, emerging evidence suggests that semaglutide possesses anti-inflammatory properties by modulating the AMPK/SIRT1 signaling pathway and inhibiting NF-κB-mediated inflammatory responses, thereby reducing obesity-associated chronic low-grade inflammation and improving metabolic homeostasis.^[21-23]^

The therapeutic efficacy and adverse effect profile of semaglutide are closely related to its pharmacological mechanism of action. As a long-acting glucagon-like peptide-1 receptor agonist, semaglutide activates GLP-1 receptors in the hypothalamus and brainstem, leading to enhanced satiety, reduced appetite, decreased food cravings, and lower caloric intake. In addition, semaglutide delays gastric emptying and suppresses glucagon secretion while enhancing glucose-dependent insulin release, collectively improving metabolic homeostasis and facilitating clinically meaningful weight reduction.^[21,22]^ The higher incidence of gastrointestinal adverse events observed in the present analysis is consistent with these physiological effects. Delayed gastric emptying and stimulation of central GLP-1 receptor pathways involved in nausea and emesis contribute to the development of nausea and vomiting, whereas altered gastrointestinal motility may lead to diarrhea or constipation, particularly during the dose-escalation phase of treatment.^[23,24]^ These adverse events are generally mild to moderate, transient, and tend to diminish with continued therapy, supporting the recommendation for gradual dose escalation to improve treatment tolerability.^[18]^ Serious adverse events, including acute pancreatitis and cholelithiasis, were uncommon across the included trials. Cholelithiasis may occur secondary to rapid weight loss and impaired gallbladder motility associated with GLP-1 receptor activation, whereas the relationship between semaglutide and acute pancreatitis remains uncertain, with current evidence suggesting a low absolute risk in appropriately selected patients.^[25]^ Overall, the substantial weight-loss benefits of semaglutide appear to outweigh its predominantly mild and manageable adverse effects, although careful patient selection, dose titration, and clinical monitoring remain essential during therapy.

The increased incidence of gastrointestinal adverse events observed in the present meta-analysis is consistent with the established pharmacological effects of GLP-1 receptor agonists. Delayed gastric emptying and direct activation of central GLP-1 receptors involved in emetic pathways contribute to nausea and vomiting, whereas alterations in gastrointestinal motility may result in diarrhea or constipation. These adverse events typically occur during dose escalation, are generally mild to moderate in severity, and tend to diminish over time as physiological adaptation develops. Consequently, gradual dose escalation has become the standard strategy to improve treatment tolerability and minimize treatment discontinuation while maintaining the substantial weight-loss benefits of semaglutide.^[18,24,25]^

Regarding safety, gastrointestinal adverse events, including nausea, vomiting, diarrhea, and constipation, were the most commonly reported complications. These adverse events were generally mild to moderate in severity, transient, and manageable without specific intervention. Nevertheless, they contributed to a significantly higher rate of treatment discontinuation in the semaglutide group. Clinicians should therefore balance the substantial weight-loss benefits against the potential for gastrointestinal intolerance and counsel patients appropriately before treatment initiation. Serious adverse events, including acute pancreatitis and cholelithiasis, were uncommon, although continued post-marketing surveillance and long-term safety studies remain warranted.

An important consideration is that semaglutide was administered alongside lifestyle interventions, including calorie restriction and increased physical activity. Consequently, the observed treatment effect likely reflects the combined benefits of pharmacotherapy and behavioral modification rather than semaglutide alone. This observation reinforces current guideline recommendations that pharmacological therapies should complement, rather than replace, comprehensive lifestyle management strategies.

## 5. Strengths and limitations

This systematic review and meta-analysis provides a comprehensive evaluation of the efficacy and safety of subcutaneous semaglutide for weight management in nondiabetic individuals with obesity by synthesizing evidence from randomized controlled trials. The review assessed clinically relevant outcomes, including percentage weight loss, gastrointestinal adverse events, treatment discontinuation due to adverse events, and serious adverse events, thereby offering a balanced evaluation of both efficacy and safety. Furthermore, the inclusion of randomized controlled trials enhances the overall quality of the evidence and strengthens the validity of the findings.

However, several limitations should be considered. First, only 4 randomized controlled trials met the eligibility criteria, limiting the overall evidence base and restricting the ability to perform subgroup analyses, meta-regression, or robust exploration of heterogeneity. Second, although extensive database searches were conducted, the possibility of retrieval bias cannot be completely excluded. Third, the review protocol was not prospectively registered in PROSPERO or another systematic review registry, which may affect methodological transparency. Fourth, ongoing studies and unpublished data were not included because complete outcome data were unavailable. Given the small number of included studies and the tendency for positive obesity pharmacotherapy trials to be preferentially published, publication bias cannot be excluded, and formal assessment of publication bias was not feasible. Additionally, the inclusion of weight data from participants lost to follow-up may have influenced pooled estimates. Clinical and methodological heterogeneity among studies, including differences in semaglutide dose, treatment duration, baseline BMI, obesity severity, participant demographics, adherence rates, behavioral support intensity, and lifestyle intervention protocols, may also have affected the observed treatment effects. Finally, the relatively short follow-up periods of the included trials limit conclusions regarding long-term efficacy, safety, and weight regain following treatment discontinuation. Despite these limitations, the available evidence consistently supports the efficacy of semaglutide in achieving clinically meaningful weight loss in nondiabetic individuals with obesity.

## 6. Conclusion

In summary, subcutaneous semaglutide was associated with substantial weight loss in nondiabetic individuals with obesity, resulting in an average weight reduction of 11.85% compared with placebo across the included randomized controlled trials. Semaglutide treatment was associated with a higher incidence of gastrointestinal adverse events and treatment discontinuation due to adverse events, although serious adverse events were uncommon. While these findings demonstrate the potential of semaglutide as an effective pharmacological intervention for weight reduction, they should be interpreted in the context of the relatively limited number of available studies and follow-up durations. The present review did not evaluate cost-effectiveness, comparative effectiveness, long-term adherence, or real-world clinical outcomes. Therefore, further large-scale randomized and real-world studies with longer follow-up periods are needed to better characterize the long-term efficacy, safety, durability of weight loss, and the potential for weight regain following treatment discontinuation. Additionally, future studies should include more diverse populations to improve the generalizability of findings across different racial and ethnic groups.

## Author contributions

**Conceptualization:** Faryal Naz, Siraj-ud-Din, Amjad Mustafa, Jahanzeb Malik.

**Data curation:** Amjad Mustafa.

**Formal analysis:** Abida Perveen.

**Investigation:** Fawad Qaiser, Amjad Mustafa.

**Methodology:** Siraj-ud-Din, Asif Ullah.

**Project administration:** Fawad Qaiser.

**Software:** Abida Perveen.

**Supervision:** Faryal Naz, Fawad Qaiser, Arif Mumtaz, Asif Ullah.

**Validation:** Arif Mumtaz, Siraj-ud-Din.

**Visualization:** Fawad Qaiser, Arif Mumtaz, Siraj-ud-Din, Asif Ullah.

**Writing – original draft:** Faryal Naz, Fawad Qaiser, Arif Mumtaz, Siraj-ud-Din, Amjad Mustafa, Asif Ullah, Abida Perveen.

**Writing – review & editing:** Faryal Naz, Fawad Qaiser, Arif Mumtaz, Siraj-ud-Din, Amjad Mustafa, Asif Ullah, Abida Perveen, Jahanzeb Malik.
